# Electro-Hydrodynamic Drop-on-Demand Printing of Aqueous Suspensions of Drug Nanoparticles

**DOI:** 10.3390/pharmaceutics12111034

**Published:** 2020-10-29

**Authors:** Ezinwa Elele, Yueyang Shen, Rajyalakshmi Boppana, Afolawemi Afolabi, Ecevit Bilgili, Boris Khusid

**Affiliations:** Otto H. York Department of Chemical and Materials Engineering, New Jersey Institute of Technology, Newark, NJ 07102, USA; eoe4@njit.edu (E.E.); sa982@njit.edu (Y.S.); rb278@njit.edu (R.B.); aa266@njit.edu (A.A.); bilgece@njit.edu (E.B.)

**Keywords:** precision dosage form, drop-on-demand printing, poorly water-soluble drugs, nanoparticles, biocompatible films, drug release profile

## Abstract

We demonstrate the ability to fabricate dosage forms of a poorly water-soluble drug by using wet stirred media milling of a drug powder to produce an aqueous suspension of nanoparticles and then print it onto a porous biocompatible film. Contrary to conventional printing technologies, a deposited material is pulled out from the nozzle. This feature enables printing highly viscous materials with a precise control over the printed volume. Drug (griseofulvin) nanosuspensions prepared by wet media milling were printed onto porous hydroxypropyl methylcellulose films prepared by freeze-drying. The drug particles retained crystallinity and polymorphic form in the course of milling and printing. The versatility of this technique was demonstrated by printing the same amount of nanoparticles onto a film with droplets of different sizes. The mean drug content (0.19–3.80 mg) in the printed films was predicted by the number of droplets (5–100) and droplet volume (0.2–1.0 µL) (*R*^2^ = 0.9994, *p*-value < 10^−4^). Our results also suggest that for any targeted drug content, the number-volume of droplets could be modulated to achieve acceptable drug content uniformity. Analysis of the model-independent difference and similarity factors showed consistency of drug release profiles from films with a printed suspension. Zero-order kinetics described the griseofulvin release rate from 1.8% up to 82%. Overall, this study has successfully demonstrated that the electro-hydrodynamic drop-on-demand printing of an aqueous drug nanosuspension enables accurate and controllable drug dosing in porous polymer films, which exhibited acceptable content uniformity and reproducible drug release.

## 1. Introduction

The precision medicine approach uses methods of molecular analysis to tailor the specific drug and dose strength to a patient’s genetic background [1,2]. The transformation of health care from one-size-fits-all to a targeted approach utilizing each patient’s molecular information is accelerating as the U.S. Food and Drug Administration (FDA) continues to rapidly approve new precision diagnostic tools and treatments. Following the approval of eleven new precision medicines by the FDA in 2019, precision medicines now account for more than one out of every four drugs approved since 2014 [3]. This is a significant increase from 2005, when precision medicines accounted just for five percent of new therapies approved by the FDA each year [3]. However, the conventional large-scale pharmaceutical manufacturing systems cannot cost-effectively address the needs of individual patients. Development of new production platforms and technologies capable of tailoring products to the individual patient’s needs is required to expand the frontiers of precision medicine. Key challenges that the pharmaceutical products, manufacturing systems, drug supply chain, and healthcare professional would have to address in order to support individualization of dosage forms were thoroughly discussed in [4,5,6,7,8].

Additive manufacturing techniques, often referred to as 3D printing, represent a new engineering approach that is expected to implement the concept of precision medicine via on-demand fabrication of dosage forms with individually adjusted doses [4,5,6]. The first printed medicine, which the FDA approved in 2015, was Spritam^®^, an epilepsy drug levetiracetam that belongs to Class I of Biopharmaceutical Classification System (BCS) [4]. Designed to disintegrate instantly after ingestion, Spritam^®^ tablets are fabricated using the ZipDose^®^ printing technology developed by Aprecia Pharmaceuticals, Blue Ash, OH [9]. In this process, drug particles are spread into thin layers, which is followed by selective jetting to bind the particles into thin porous layers. Depending on the required dose strength, printing is repeated for a specific number of times to build the product layer by layer.

Following the FDA approval of ZipDose^®^ technology, printing, both 2D and 3D, has gained momentum in pharmaceutics for the manufacturing of precision dosage forms tailored to individual patient needs. Computer-controlled noncontact drop-on-demand (DOD) printing, in which droplets are formed only as required, is regarded as one of the most promising techniques for small-scale manufacturing of precision medicines. Depending on the utilized print heads, the well-studied nature of the jetting processes allows for different pharmaceutical materials to be printed onto the same object. DOD printing of various pharmaceutical formulations, such as liquid solutions, emulsions, and suspensions of drug micro- and nanoparticles, lipid vesicles, proteins, and cells was thoroughly reviewed in [4,5,6,7,8,10,11,12,13,14,15]. The driving factors for the DOD printing include flexibility to compound drugs and vary the dose strength, potential for low-cost production of small quantities of precision products, and ability to build on-demand production of medicines at the point-of-care and onsite in remote locations [4,5,6]. Moreover, printing technology is expected to address today’s challenges involved with transforming poorly water-soluble drugs into viable medicinal products [10]. Printing of precision dosage units is considered to reduce drug waste and achieve a desired drug release profile by printing a vast array of therapeutics in a specific pattern on a biocompatible film. Porous films can be used to facilitate absorption of deposited droplets and thereby improve printing accuracy [11,12]. Printed films can then be folded and assembled like origami into three-dimensional structures to reduce printing time by reducing the number of printing layers and eliminating the need for support material [6]. Printing technologies can also provide patients with the ability to vary characteristics of their individual medicines, such as taste-masking, shape, color, and embossment, and even to combine different medicines in the same dosage form. The patients’ perceptions, preferences, and acceptability of all these options were explored in the recent pilot study [13]. Providing patients with the ability to design individual medicines can facilitate patient engagement and lead to improvements in patient care and health outcomes [7,13].

Although promising, the application of DOD printing to pharmaceutical products poses a challenge [14,15]. There are many technical and regulatory questions that need to be addressed before this technology moves on to widespread pharmaceutical applications. One of them is the need to develop a versatile printing technique due to the limited capabilities of commercially available printing systems. Designed mainly for printing of images, conventional print heads employ a short pressure pulse generated thermally or piezoelectrically or mechanically to expel a droplet out of a nozzle [16,17]. In the thermal method, a small volume of liquid is vaporized by heating up to 200–300 °C to form a bubble that expands and ejects a droplet. In the piezoelectric and electrostatic methods, a rapid change in the shape of a piezoelectric crystal or an electrically driven mechanical displacement are utilized to apply a strong pressure impulse directly to an ink-filled nozzle in order to eject a droplet by exposing the ink to high shear rates of up to $10^{5}s^{-1}$. Conventional print heads are typically designed for a particular ink to produce droplets with a narrow size distribution. The droplet size is governed by characteristics of the printing regime and the ink viscosity and surface tension. Therefore, the same print head cannot be used for dosing pharmaceutical formulations with widely different properties or via droplets of different sizes. The working principles of inkjet printing have been developed for chemically and thermally stable, low-viscosity inks typically in the range 1–30 mPa·s because of pressure pulse limitation [16,17]. Therefore, conventional inject systems appear to be of limited use for pharmaceuticals due to fundamentally different functional requirements.

A pharmaceutical product is a unique, delicate formulation composed of active pharmaceutical ingredients (APIs) and inactive excipients to ensure a desired absorption rate in the human body and stability over long-term storage. It has to be protected against chemical changes, which can occur in print heads due to excessive heating or high shear stress during droplet deposition. Next, DOD systems for pharmaceutical applications should be able to print products having different physical properties over a broad range of droplet sizes with high volume accuracy. The present work reports a DOD technique for fabrication of dosage forms by encapsulating nanoparticles of a poorly water-soluble drug into a biocompatible porous film. A porous film is used to facilitate the absorption of the deposited droplets. Experiments were conducted with the electrodeless electro-hydrodynamic (EHD) DOD method developed in Ref. [18] for gentle dispensing of highly viscous liquids. This method provides a precise control over a liquid volume, since the liquid properties and characteristics of a printing regime do not affect the droplet size. To eliminate the adverse effects of electro-chemical reactions at the liquid-electrode interface in the course of printing, a liquid specimen does not come directly into contact with electrodes. The method versatility was proven on liquids spanning over three orders of magnitude in viscosity (1–3112 mPa·s) over the droplet size ranging from 0.1 to 2 μL [18]. A nozzle heater was used to dispense materials with a melting point above room temperature. Results of theoretical and experimental study [18] provide the relationship for the selection of a printing regime based on the material’s properties. As there are no limitations on the extent to which one can vary the liquid viscosity and the droplet size, the EHD DOD dispensing offers a powerful tool for fabrication of dosage forms by encapsulating an API into a biocompatible porous film that can be covered with a non-porous barrier film on one or two sides.

The use of the EHD DOD method for pharmaceutical applications was tested in [19] by printing polyethylene glycol (PEG), 400 solutions of poorly water-soluble ibuprofen (a non-steroidal anti-inflammatory drug), and griseofulvin (an antifungal drug) onto a porous hydroxypropyl methylcellulose (HPMC) film prepared by freeze-drying. Viscosities of these solutions exceeded the operating range of conventional inkjet print heads by a factor of 15–20. As the printed drug solution oversaturated with time due to mixing with HPMC, ibuprofen and griseofulvin formed amorphous precipitates encapsulated into the film. The accuracy of positioning printed droplets was 1.2%. Dose-to-dose variability was found to decrease rapidly with increasing the number of droplets from 4–9% for 5 droplets to 1–2.4% for 40 droplets. 112 droplets were printed to produce a drug load of about 9.4 (*w/w*) % per assembled dosage unit. The reproducibility of the EHD DOD printing was shown by the comparison of the drug release profiles from six films, each with 112 printed droplets, to one another, according to the basic FDA guidelines [20].

Unlike in our previous studies [18,19], here we use the EHD DOD method for printing aqueous suspensions of drug nanoparticles, also known as drug nanosuspensions. Drug nanosuspensions have been widely used for improving the dissolution rate and bioavailability of poorly water-soluble drugs owing to the large surface area of drug nanoparticles [21,22,23]. Drug nanosuspensions are typically dried into nanocomposite powders that are further processed into solid dosage forms because of better patient compliance with and convenience of solid dosages as opposed to suspensions (see [24] and the references cited therein). Standard drying methods such as spray drying [25,26], fluidized bed coating/drying [27,28], and freeze drying [29,30] are not conducive to the preparation of precision dosage forms. Investigation of the EHD DOD printing of drug nanosuspensions is warranted, as it offers the promise of producing uniform precision dosages containing drug nanoparticles. In contrast to tests in [19], dissolution of a drug in a carrier liquid is not required, and the particles retain their crystallinity and polymorphic form during nanosuspension preparation and printing.

The aim of this study is to examine the accuracy of EHD DOD printing of a drug nanosuspension onto porous polymeric films and assess dose-to-dose reproducibility of the drug release kinetics during in vitro dissolution tests. Griseofulvin was taken as a representative model of BCS Class II drugs due to its low water solubility of approximately 12 mg/L [31,32]. The aqueous nanosuspension of griseofulvin was selected as a viable choice for delivery of BCS Class II drugs, because it is environmentally benign, operationally safe, and provides an extremely large surface area for drug release [23,24]. Griseofulvin nanoparticles were prepared using wet stirred media milling, which has been proven as a robust top-down process for producing stable suspensions of drug nanoparticles [33,34]. Composition of griseofulvin suspensions and processing parameters of wet milling were selected following refs. [35,36,37]. A desired spatial pattern of griseofulvin was produced by printing droplets from a nozzle onto a porous hydroxypropyl methylcellulose (HPMC) film in specific locations that then spread over and imbibed into the film. Number–volume of droplets were varied to print 0.19–3.80 mg drug. Drug content and its uniformity was determined by UV spectroscopy. Differential scanning calorimetry (DSC) and X-Ray diffractometry were used to examine crystallinity changes in griseofulvin that may result from milling and printing. Drug release kinetics was investigated using a Flow-Through Cell Dissolution Apparatus. The reported technique can be used to form a precision dosage unit by combining several films with the same or different APIs. In particular, films can form a therapeutic dosage by scoring, cutting, and folding together or placing into a capsule shell.

## 2. Materials and Methods

This section describes the materials used in the EHD DOD printing process and experimental procedures used for the preparation and characterization of the griseofulvin nanosuspensions as well as characterization of the dosage form and parameters of the printing regime.

### 2.1. Materials

Griseofulvin powder (BP/EP micronized) was purchased from Letco Medical (Decatur, AL, USA). Sodium dodecyl sulfate (SDS) powder (for molecular biology, ≥98.5% GC) obtained from Sigma-Aldrich (St. Louis, MO, USA) was used as a wetting agent in griseofulvin suspensions and in dissolution testing. Hydroxypropyl methylcellulose (HPMC) Methocel E15LV powder was purchased from Dow Chemical (Midland, MI, USA). Known for its good film-forming property and biocompatibility, HPMC was used as a steric stabilizer in griseofulvin suspensions and in preparing porous films.

### 2.2. Preparation of Suspensions

Table 1 presents the compositions of two aqueous griseofulvin suspensions used in experiments. Throughout the paper, all percentages (%) in a suspension composition refer to *w/w* with respect to de-ionized (DI) water. These suspensions were produced by utilizing HPMC concentrations (1.5% and 2.5%) along with 0.5% SDS as stabilizers during the wet stirred media milling. Milling for 64 min reduced the median size of particles from 20 µm to about 160 nm.

The wet milling regimes and stabilizers for producing suspensions were selected based on previous studies [35,36,37,38]. A feed for wet milling was prepared by using a shear mixer (DLM 1638 × 1, Fisher Scientific, Pittsburgh, PA, USA) running at a fixed speed of 300 rpm. The HPMC and SDS powders were dispersed in DI water for 60 min followed by dispersion of the griseofulvin powder for 30 min. Nanoparticles were produced by milling a feed (40 g griseofulvin per 200 g DI-water) in a Microcer wet-stirred media mill (Netzsch Fine Particle Technology, LLC, Exton, PA, USA) operating in the recirculation mode. Wear-resistant yttrium stabilized zirconia beads (Zirmil Y, Saint-Gobain ZirPro, Mountainside, NJ, USA) with a median size of 430 µm were used as the milling media. A 200-m screen kept the beads in the milling chamber of 80 mL. The feed was poured into the holding tank and milled for 64 min under the following conditions: a load of the milling chamber with beads was 50 mL, the recirculating flow rate of the feed between the holding tank and the milling chamber was 126 mL/min, and the rotor speed was 3200 rpm that corresponded to a tip speed of 11.7 m/s. The prepared griseofulvin suspensions were stored at 8 °C in a refrigerator. The size change of nanoparticles, D50, following a storage period of 7 days lay within measurement errors reported in Table 1.

### 2.3. Characterization of Suspensions

All measurements were performed within 7 days after preparation of a suspension. The size distribution of griseofulvin particles in suspensions was measured on a Beckman Coulter LS 230 (Brea, CA, USA) equipped with a polarization intensity differential scattering (PIDS) module. The PIDS ranged between 40% and 50%, and the specimen obscuration was maintained below 8%. The scattered light was analyzed by the Mie optical model with water as solvent. Refractive indices 1.65 and 1.33 were, respectively, taken for griseofulvin [38] and DI water. Prior to measuring the particle size, a suspension specimen of about 2 mL was diluted with 10 mL of the respective HPMC-SDS solution.

**Figure 1:**
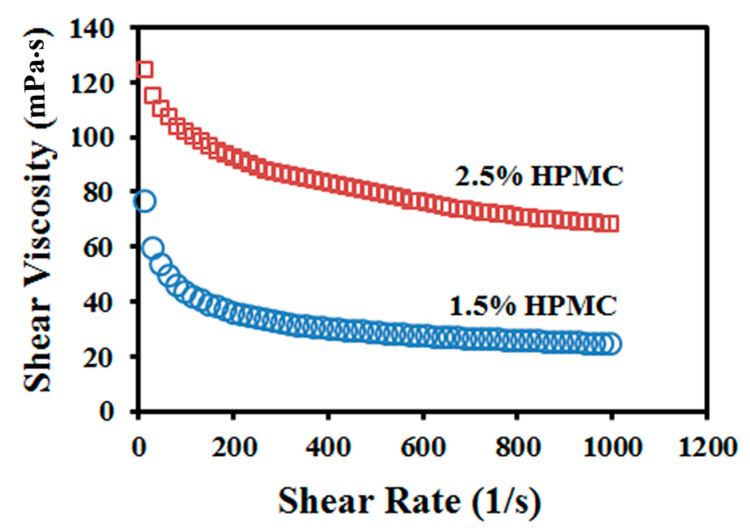
Suspension shear viscosity vs. shear rate.

The apparent shear viscosity of griseofulvin suspensions was measured on an R/S Plus Rheometer (Brookfield Engineering, Middleboro, MA, USA). A coaxial cylinder was used to impart a shear rate from 0 to 1000 s^−1^ in 60 s on a specimen. The temperature of the jacket was kept constant at 25 ± 0.5 °C. The suspension density was found by weighing a specimen filled in a 50-mL volumetric flask. A Sigma Force Tensiometer (Attension, KSV Instrument, Espoo, Finland) was used to measure the suspension surface tension. The respective suspension (see Table 1) was poured into a 50-mL calibrated beaker with a diameter of 46 mm, and the surface tension was determined through the force exerted by the liquid meniscus on the Wilhelmy plate (19.6 mm × 10 mm and thickness of 0.1 mm, wetted length and depth 39.4 mm and 6 mm, respectively, Attension, KSV Instrument, Espoo, Finland) moving at 20 mm/min. The shear rate dependence of the suspension viscosity is shown in Figure 1. The drug nanosuspensions exhibit pseudoplastic (shear-thinning) behavior. Values for the shear viscosity reported in Table 1 are taken for shear rate 1000 s^−1^.

**Figure 2:**
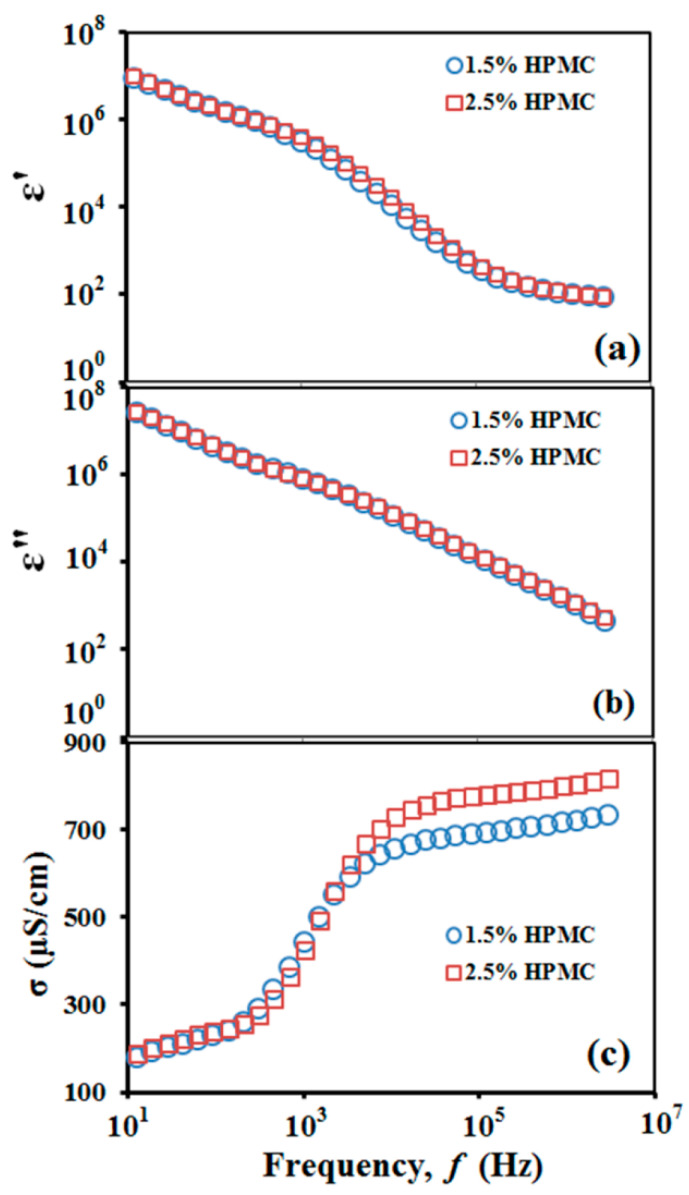
Frequency dependence of the suspension electric properties: (**a**) real part of complex permittivity ɛ’; (**b**) imaginary part of complex permittivity ɛ”; (**c**) specific conductivity $\sigma =2{\pi f\varepsilon }_{0}\varepsilon ”$, where f is the field frequency in Hz and $\varepsilon_{0}=8.85\times 10^{-12}F/m$ is the vacuum permittivity.

Electric conductivity and dielectric constant of griseofulvin suspensions were measured on a Broadband Dielectric Spectrometer (BDS)-80 (Novocontrol, Hundsangen, Germany). Measurements were conducted over the frequency range 1 Hz–3 MHz on a 0.8-mL specimen poured in a BDS sample cell for liquids with high permittivity and ion conductivity. The frequency dependence of the suspension electric properties is shown in Figure 2. Values for electric characteristics reported in Table 1 are taken for frequencies above 10 kHz, where they become frequency independent as the electrode polarization is suppressed.

### 2.4. Preparation of Porous HPMC Films

Porous HPMC films were fabricated by freeze-drying of a 10-wt% HMPC solution. Following the Dow Chemical protocol [39], the solution was prepared by mixing a weighed amount of HPMC powder with hot DI water to form a visually homogeneous, transparent liquid and then slowly cooled down to room temperature while stirring. The HPMC solution was poured into a well formed by a rubber ring that was placed on a glass slide coated with a fluoropolymer release liner (3M, St. Paul, MN, USA). The well was covered with another glass slide (coated with the same liner) and kept in a freezer for 12 h. Then it was transferred to a freeze drier (Labconco, Kansas City, MO, USA) and kept for 24 h to sublimate water, leaving a porous film. The prepared films were stored in a desiccator at room temperature.

### 2.5. EHD DOD Printing

**Figure 3:**
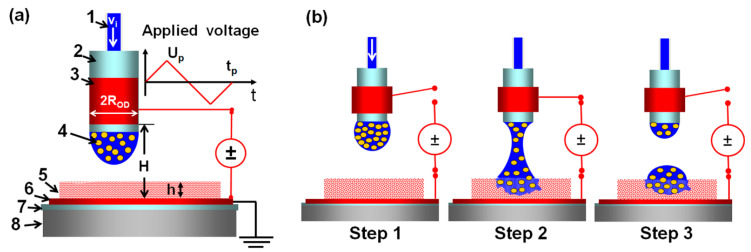
EHD DOD concept: (**a**) printing device: 1, infused fluid volume v_i_; 2, insulating nozzle; 3, energized electrode; 4, suspension, 5, porous polymer film; 6, insulator; 7, ground electrode; 8, 3D-movable stage; 2R_OD_, the nozzle outer diameter; H, the separation between the electrodes; h, the film thickness, and U_p_, t_p_ are the peak and length of a voltage pulse applied to the electrodes. (**b**) Three-step printing process: (1) a fluid volume is infused into the nozzle to form a pendant droplet, (2) an electric force generated by an applied voltage stretches the droplet to form a liquid bridge anchored to the nozzle exit and the film, (3) the liquid bridge breaks up, creating a sessile droplet on the film and the other hanging from the nozzle. Adapted with permission from [19], Elsevier, 2012.

A concept of the EHD DOD printing is illustrated in Figure 3.

Technical details of the printing device and operation were reported in Refs. [18,19]. A porous HPMC film was placed on the ground electrode mounted on a three-dimensional movable stage of a micromanipulator (Figure 3a). The micromanipulator was used to position the insulating nozzle over a desired film location. A predefined suspension volume was infused into the nozzle to form a pendant drop whose size was smaller than the critical size for dripping (step 1 in Figure 3b). A short voltage pulse was then applied to the energized electrode wrapped around the nozzle to stretch the droplet by an electric force until it touched the HPMC film and formed a liquid bridge anchored by capillary forces to the nozzle exit and the film (step 2 in Figure 3b). During the pulse, the droplet served as a floating electrode capacitively coupled to the energized and ground electrodes, as it did not contact them directly. The pulse consisted of two symmetric, phase-alternating signals to suppress undesirable electric field effects on the droplet (Figure 3a). The liquid bridge broke up, creating two droplets, one on the film and the other hanging from the nozzle (step 3 in Figure 3b). Once formed, the sessile droplet spread over and imbibed into the porous film. The presence of an electric field was not required during this period.

The volume of a sessile droplet deposited on a film was shown to be equal to the volume infused in the nozzle, v_i_, for a steady-state printing operation [18]. A pendant drop is formed by infusing a fluid specimen into the nozzle with the flow rate and volume controlled by a programmable microsyringe pump [18,19]. In the case of an impermeable film, the magnitudes of v_i_ and the separation between the electrodes, H, should be chosen so as to form an unstable liquid bridge in step 2 (Figure 3) that would break up and create a sessile droplet in step 3 (Figure 3). As was demonstrated in Ref. [18], the calculated stability limit of an equilibrium liquid bridge anchored by capillary forces to unequal coaxial disks at negligible gravity provided the maximum droplet volume, v_i_, as a function of H that would form an unstable liquid bridge. If the droplet size exceeds this limit, the bridge would not break up. However, it was shown that larger droplets could be printed on a porous film at the given separation between the electrodes, H, as a liquid bridge that would eventually become unstable when its volume decreased below the stability limit due to fluid imbibition into the porous film. Printing was performed within 5 days after the preparation of a suspension.

### 2.6. Characterization of HPMC Film with Printed Suspension Droplets

After printing, the films with printed droplets were stored overnight in a desiccator at room temperature, and their properties were measured the next day.

#### 2.6.1. Differential Scanning Calorimetry (DSC)

DSC curves of specimens were recorded on a Mettler Toledo Polymer DSC (Columbus, OH, USA) calibrated with a Mettler Toledo indium sample. Measurements were conducted on a 3.5-mg porous HPMC film with 5 printed 0.5-µL suspension droplets (calculated griseofulvin content 0.45 mg). For comparison, measurements were also carried out on a porous HPMC film (3 mg) without the suspension and as-received griseofulvin powder (0.5 mg). Specimens were heated at a rate of 5 °C/min over the temperature range of 200–240 °C to cover the melting point of as-received griseofulvin powder.

#### 2.6.2. X-Ray Diffractometry (XRD)

Diffractograms of specimens were collected on a PANalytical Empyrean Series 2 X-Ray Diffractometer (Westborough, MA, USA) equipped with a Cu Kα X-ray source. The angular range (θ) from 8° to 36° was recorded with a step-width of 0.007° and a scan speed of 0.164° per second. Measurements were carried out on a 20-mg porous HPMC film with 50 printed 0.5-µL suspension droplets (calculated griseofulvin content 4.51 mg). Comparison was made with XRD patterns of a porous HPMC film (20 mg) without the suspension and as-received griseofulvin powder (4.5 mg). The data were analyzed using the griseofulvin XRD pattern stored in the instrument library (Reference code: 00-040-1937).

#### 2.6.3. Measurement of Griseofulvin Content

Measurements were conducted at room temperature. An HPMC film with printed droplets was placed in a stirring beaker and dissolved in 100 mL of a 5.4-g/L (18.7 mM) SDS solution in DI-water to form a transparent liquid. The SDS concentration was taken well above the micellar point to increase the griseofulvin solubility up to 560 mg/L estimated based on data [31] or 430 mg/L based on data [32]. The griseofulvin concentration in micellar SDS solutions was measured by UV spectroscopy in Ref. [31] and by high-pressure liquid chromatography in Ref. [32]. Following [31], we employed UV spectroscopy for measuring the griseofulvin content.

Absorbance at a wavelength of 293 nm was measured with an Evolution 300 UV-Vis spectrophotometer (Thermo Fisher Scientific, Waltham, MA) in 3.5-mL quartz cuvettes (Cole-Parmer, Vernon Hills, IL). Preliminary experiments showed that the presence of HPMC in the solution over the range of tested compositions did not affect the absorbance value. A griseofulvin suspension with 1.5% HPMC (Table 1) was diluted with a 5.4-g/L SDS solution in DI-water to prepare solutions with griseofulvin concentrations of 1.8, 3.6, 7.3, 14.5, 21.8, and 36.3 mg/L that were used to construct a calibration curve. A plot of absorbance vs. griseofulvin concentration demonstrated the linearity over the range of expected concentrations with a determination coefficient *R*^2^ of 0.9998.

#### 2.6.4. Thermo-Gravimetric Analysis (TGA)

TGA curves of specimens were recorded on a thermo-gravimetric analyzer Mettler-Toledo TGA/DSC1/SF Stare system (Mettler Toledo, Inc., Columbus, OH, USA). Measurements were conducted on three (2.5 ± 0.3)-mg porous HPMC films, each with 5 printed 0.5-µL suspension droplets (calculated griseofulvin content 0.45 mg), following overnight storage in a desiccator at room temperature. For comparison, measurements were also carried out on three (2.5 ± 0.3)-mg porous HPMC films without the suspension. A specimen was placed in a ceramic crucible, heated from room temperature (25 °C) to 150 °C in nitrogen atmosphere at a rate of 5 °C/min, kept at 150 °C for 15 min, and then heated to 200 °C at a rate of 5 °C/min. Finally, the specimen was brought back to room temperature at a cooling rate of 10 °C/min.

#### 2.6.5. Dissolution Tests

Comparison of commonly-used dissolution apparatuses (the paddle, rotating basket, and flow-through cell) demonstrated that the flow-through cell method was unequivocally the most robust technique for testing drug nanoparticles [40,41]. Therefore, dissolution tests of HPMC films with printed droplets were carried out on a Flow-Through Cell Dissolution Apparatus (USP apparatus 4, Sotax, Switzerland) equipped with seven 19-mL sample cells of 22.6 mm inside diameter. A 5-mm ruby bead was positioned at the base of the sample cell and 3 g of 1-mm glass beads (Worf Glaskugeln, Germany) were placed to fill its bottom conical part. A film specimen was situated horizontally on the glass beads. Following [40,42], a 25-mm HT Tuffryn membrane (Pall Life Sciences, NY, USA), pore size 0.2 µm, was positioned above the specimen to retain large aggregates of particles and pieces of the HPMC film, and additional 3 g of 1-mm glass beads were then placed above the membrane. Tests were conducted in a closed loop configuration at a temperature of 37 ± 0.5 °C by pumping 100 mL of a 5.4-g/L SDS solution in DI-water through each cell at a flow rate of 16 mL/min; six cells were loaded with film specimens while one cell containing only a ruby bead served as a control. The griseofulvin concentration in a solution was determined by measuring at a wavelength of 293 nm with an Evolution 300 UV-Vis spectrophotometer every 2 min. A calibration curve was constructed using solutions with griseofulvin concentrations of 3.5, 15, 30, 45, and 55 mg/L that were prepared by dilution of griseofulvin suspension with 2.5% HPMC (Table 1) with a 5.4-g/L SDS solution in DI-water. A plot of absorbance vs. griseofulvin concentration demonstrated the linearity with a determination coefficient *R*^2^ of 0.9972.

To quantify the effects of the HPMC film on the griseofulvin release, dissolution tests were also conducted on the griseofulvin suspension taken for printing. In this case, a proper amount of the suspension was weighed out and then carefully situated between beads in a sample cell of the dissolution apparatus. Testing was carried out using the same procedure as that for HPMC films with printed droplets.

The release profile of griseofulvin was constructed by plotting the percentage of a dissolved drug as a function of time. Release profiles from different films with printed suspension droplets were compared using the model-independent parameters f_1_ and f_2_ referred to as difference and similarity factors, respectively [40,43]:

(1) $$f_{1}=100\left[\overset{n}{\underset{j=1}{\sum }}\left|Y_{j}-T_{j}\right|/\overset{n}{\underset{j=1}{\sum }}Y_{j}\right]$$

(2) $$f_{2}=50\log\left[100\left(1+\frac{1}{n}\overset{n}{\underset{j=1}{\sum }}{\left(Y_{j}-T_{j}\right)}^{2}\right)\right]$$

where n is the number of time points, Y_j_ and T_j_ are the values of two drug release profiles at time t_j_. The factor f_1_ expressed in percentages measures the relative difference between these profiles over all time points. The factor f_2_ also expressed in percentages measures their closeness by characterizing the sum of squared differences between them with emphasis on the larger difference among all time points. Drug release profiles are considered similar when the calculated values of f_1_ fall between 0 and 15 and values of f_2_ lie within the range 50–100.

## 3. Results and Discussion

### 3.1. Processing Characteristics

Most experiments were carried out on suspensions with 2.5% HPMC (Table 1, Figure 1) to explore the ability of the EHD DOD technique to print highly viscous suspensions of nanoparticles. Suspensions were printed on circular cuts made with a 5/8” (15.88 mm) diameter punch (O’Brien Consolidated Industries, Lewiston, ME, USA) from porous HPMC films prepared by freeze-drying of an aqueous solution. Films were 0.8 ± 0.03 mm thick with porosity φ=0.92, calculated from the HPMC concentration in the solution as $\varphi =\varphi_{w}\rho_{p}/\left(\varphi_{w}\rho_{p}+\varphi_{p}\rho_{w}\right),$ where ρ_p_ = 1290 kg/m^3^ and ρ_w_ = 1000 kg/m^3^ are, respectively, the densities of HPMC and water, ${\mathrm{and}\;\varphi }_{p}=0.1$ and $\varphi_{w}=0.9$ are, respectively, the weight fractions of HPMC and water. The film thickness and porosity were selected such that droplets printed onto a film were imbibed in the pores without emerging on the opposite side.

**Figure 4:**
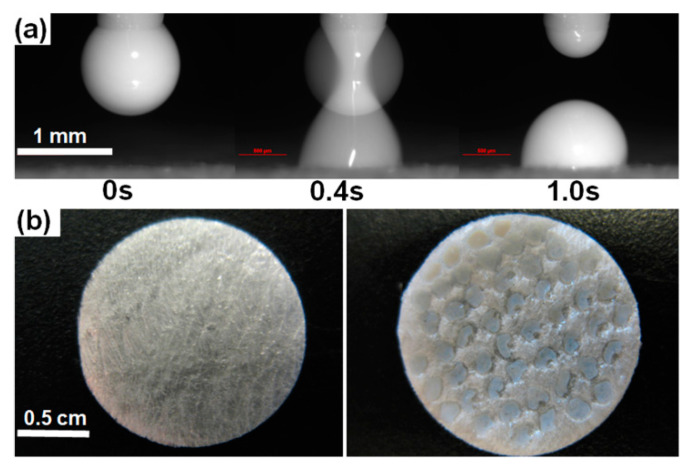
Printing 0.5-μL droplets of 2.5% HPMC-griseofulvin suspension onto 92%-porous HPMC film: (**a**) dispensing of a droplet, (**b**) 20.6-mg porous HPMC film before (*left*) and after (*right*) printing of fifty 0.5-μL droplets.

To avoid the clogging of the dispensing system, suspensions were dispensed through a Teflon nozzle with the outer diameter (OD) 0.76 mm and the inner diameter (ID) 0.56 mm (Small Parts, Logansport, IN, USA). The EHD DOD printing regime was selected based on the relationship [18] between the voltage pulse magnitude, $U_{p}$, length, $t_{p}$, and the fluid density, $\rho_{f}$, surface tension, $\gamma_{f}$, viscosity, $\eta_{f}$, nozzle radius, $R_{\mathrm{OD}}$, and inter-electrode separation, H. This relation is expressed in terms of the following non-dimensional parameters (Figure 3): the pulse electric Bond number $B_{e}=\varepsilon_{0}U_{p}^{2}R_{\mathrm{OD}}/2\gamma_{f}H^{2}$ with $\varepsilon_{0}=8.85\times 10^{-12}F/m$ being the vacuum permittivity, the Ohnesorge number $\mathrm{Oh}={\;\eta }_{f}/\sqrt{{\;\rho }_{f}R_{\mathrm{OD}}\gamma_{f}}$, the pulse impulse $I_{p}=\left(B_{e}/t_{c}\right)\int_{0}^{t_{p}}{\left(U_{e}\left(t\right)/U_{p}\right)}^{2}\mathrm{dt}$ with the capillary time $t_{c}=\sqrt{\rho_{f}R_{\mathrm{oD}}^{3}/\gamma_{f}}$ and $U_{e}\left(t\right)/U_{p}$ representing the pulse shape; $I_{p}=B_{e}t_{p}/3t_{c}$ for triangular pulses used in our experiments, Figure 3a. Based on the suspension properties reported in Table 1, we evaluated the Ohnesorge numbers and capillary times of griseofulvin suspensions and selected $B_{e}=0.5$ and $I_{p}=2.8\;$(Table 1), which yielded $U_{p}=7\mathrm{kV}$, $t_{p}=20\;\mathrm{ms}\;$for $R_{\mathrm{OD}}=0.38\;\mathrm{mm},\;H=2\;\mathrm{mm}$ Although the suspension electrical conductivity (0.7 mS/cm, Table 1) was at the level of tap water, a high-voltage pulse did not create a short circuit between the electrodes in the course of printing, as they were not in contact with the suspension (Figure 3a). Droplets were printed one by one onto a porous HPMC film. The droplet size varied in experiments from 0.2 to 1.0 µL with the critical volume of 0.3 µL above which the liquid bridge formed in step 2 (Figure 3b) at $R_{\mathrm{OD}}=0.38\;\mathrm{mm},\;H=2\;\mathrm{mm}\;$would become stable [18]. However, larger sessile droplets were formed in step 3 (Figure 3b), since the liquid bridge broke up in our experiments, as its volume was eventually reduced below the critical value due to the imbibition of the suspension by the porous film. To prevent overlapping of neighboring droplets, the spacing between centers of adjacent droplets was maintained at 2 mm for a suspension with 2.5% HPMC and at 3 mm for a suspension with 1.5% HPMC. Photos presented in Figure 4a demonstrate that dispensing of a suspension with 2.5% HPMC began with a pendant droplet, proceeded to the liquid bridge, and then to the 0.5-µL sessile droplet. Photos in Figure 4b show that a 20.6-mg 92%-porous HPMC film maintained its integrity in the course of printing fifty 0.5-µL droplets.

**Figure 5:**
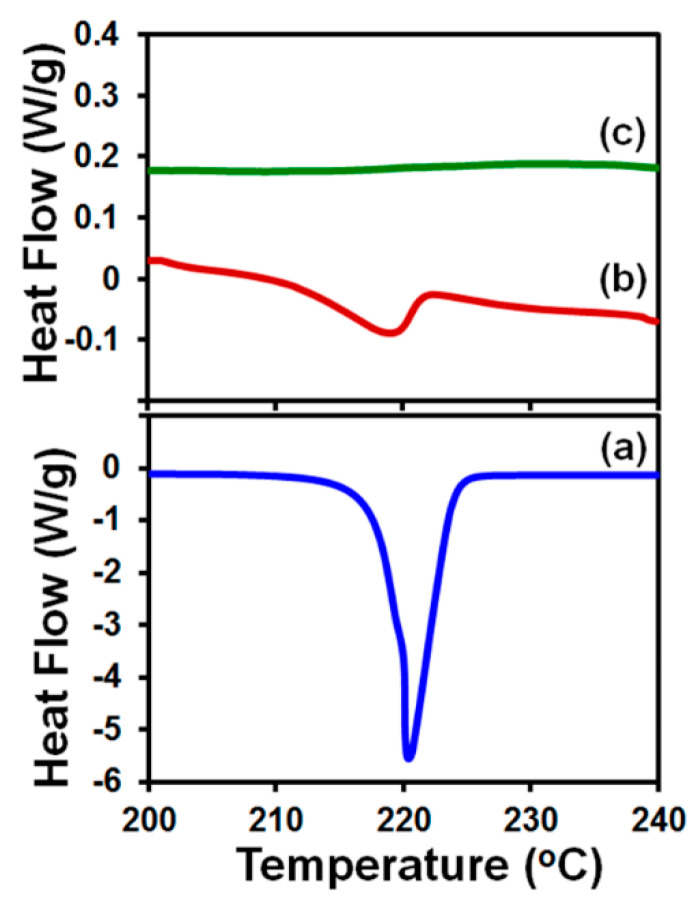
DSC curves of specimens overlaid on the temperature range 200–240 °C: (**a**) as-received griseofulvin powder (0.5 mg); (**b**) 3.5-mg porous HPMC film with five printed 0.5-µL droplets of 2.5% HPMC-griseofulvin-suspension (calculated griseofulvin content 0.45 mg); (**c**) porous HPMC film (3 mg) without the suspension.

DSC curves of 3.5-mg porous HPMC films with five printed 0.5-µL droplets of griseofulvin suspension with 2.5% HPMC (calculated griseofulvin content 0.45 mg) and, for comparison, DSC curves of 3-mg porous HPMC films without the suspension and 0.5-mg as-received powders are displayed in Figure 5 in the temperature range 200–240 °C that included the melting peak of as-received powder.

**Figure 6:**
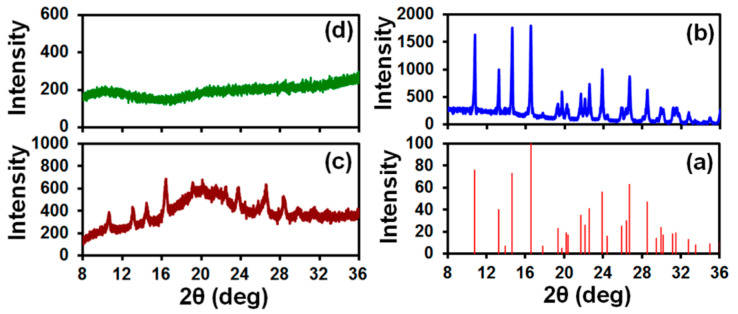
X-ray diffractograms: (**a**) griseofulvin reference pattern in the instrument library; (**b**) as-received griseofulvin powder (4.5 mg); (**c**) 20-mg porous HPMC film with 50 printed 0.5-µL droplets of 2.5% HPMC-griseofulvin suspension (calculated griseofulvin content 4.51 mg); (**d**) porous HPMC film (20 mg) without the suspension.

The onsets of the melting temperature for griseofulvin encapsulated in the film (217.8 ± 1.2 °C) and for as-received powder (218.4 ± 0.6 °C) appear to be consistent. Furthermore, crystalline peaks in the X-ray diffractogram of 20-mg porous HPMC films with fifty printed 0.5-µL droplets (calculated griseofulvin content 4.51 mg) displayed in Figure 4 agree well with the griseofulvin reference pattern in the instrument library, albeit being smaller than peaks observed in 4.5-mg as-received powder. The presented results are consistent with the characterization of griseofulvin via DSC, XRD, and Raman spectroscopy [37], which showed that wet media milling does not alter the griseofulvin crystalline state and confirm that printing maintained the particle crystallinity. The broadening of the melting endotherm and the reduction in the XRD peak heights (Figure 6) could be explained by the formation of griseofulvin nanocrystals upon milling as well as their dilution and encapsulation in the HPMC film.

**Figure 7:**
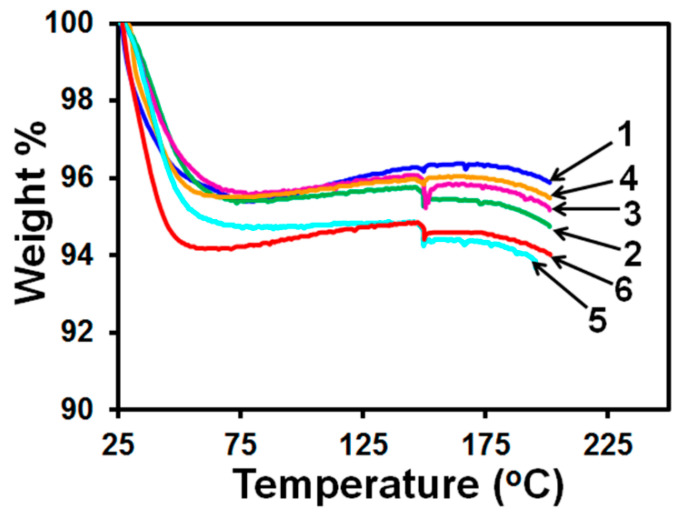
Curves of the HPMC matrix weight percentage vs. temperature for (1–3) (2.5 ± 0.3)-mg porous films with 5 printed 0.5-µL droplets of 2.5% HPMC-griseofulvin suspension (calculated griseofulvin content 0.45 mg) after overnight storage in a desiccator at room temperature and for (4–6) (2.5 ± 0.3)-mg porous films without the suspension.

TGA measurements were carried out on three (2.5 ± 0.3)-mg porous HPMC films with five printed 0.5- µL droplets of griseofulvin suspension with 2.5% HPMC (calculated griseofulvin content 0.45 mg) after overnight storage in a desiccator at room temperature and, for comparison, on three (2.5 ± 0.3)-mg porous HPMC films without the suspension. To quantify the contribution of water in the printed suspension to the HPMC matrix weight loss, the weight percentage for such specimens was computed by subtracting the weight of nanoparticles (0.45 mg) from the recorded specimen weight as $\frac{M-M_{g}}{M_{\mathrm{in}}-M_{g}}$, where M is the measured specimen weight, $M_{\mathrm{in}}$ is the initial specimen weight, and $M_{g}$ is griseofulvin weight. Curves of the HPMC matrix weight percentage vs. temperature are displayed in Figure 7. For all specimens, weight losses of 4–6 wt % occurred under heating from room temperature up to about 70 °C and were insignificant under further heating. The fact that the same trend was observed for the films with and without the suspension indicates that water imbibed by a porous film in the course of printing evaporated during the overnight storage.

### 3.2. Drug Release Profile

**Figure 8:**
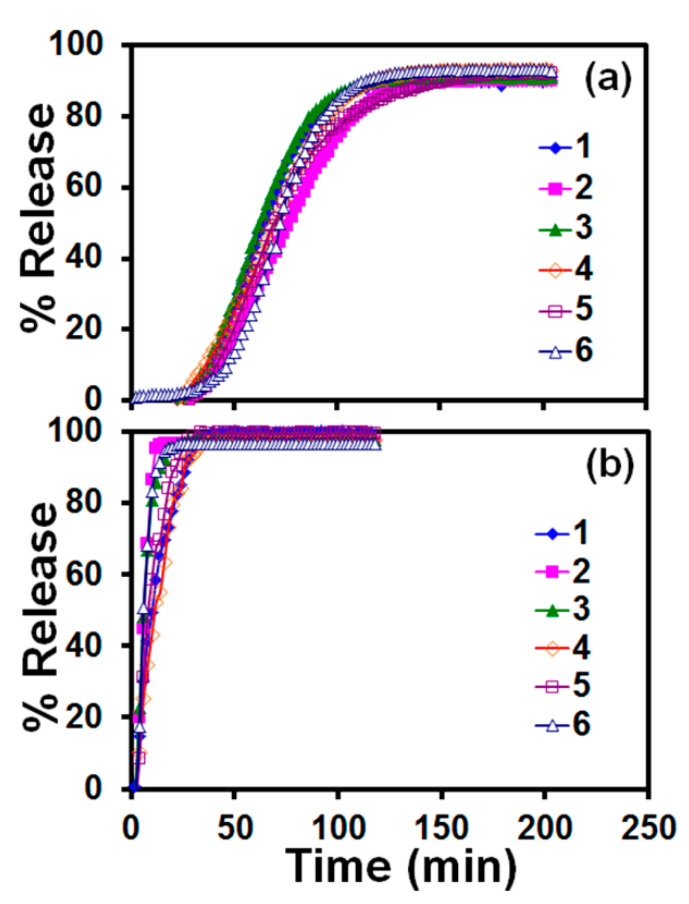
Release profiles of griseofulvin from (**a**) six porous (20.4±0.4)-mg HPMC films, each loaded with 25 µL of 2.5% HPMC-griseofulvin suspension by printing fifty 0.5-µL droplets, and (**b**) six 25-µL specimens of the same griseofulvin suspension.

To measure the release of griseofulvin from nanoparticles encapsulated in the HPMC film, we prepared six (20.4±0.4)-mg porous HPMC films, each loaded with 25 µL of griseofulvin suspension with 2.5% HPMC by printing fifty 0.5 µL-droplets. The release of griseofulvin from these films was then compared with its release directly from six 25-µL specimens of the same suspension. Measurements are plotted in Figure 8.

The calculated griseofulvin content of each specimen displayed in Figure 8 was 4.51 mg. The peak value of the griseofulvin release averaged over six profiles reached 90.5±1.6% (mean ± standard deviation) in 122 min for the film specimens (with a *p*-value of 0.106 at 95% confidence) and 97.8±1.2% in 26 min for the suspension specimens (with a *p*-value of 0.176 at 95% confidence level). Common models [44,45] were tested to describe the release profiles plotted in Figure 6. The equation for zero-order release kinetics $Q_{t}=K_{\mathrm{film}}\left(t-t_{0,\mathrm{film}}\right)$, where $Q_{t}$ is the cumulative amount (in %) of griseofulvin released at moment t (in min) with $K_{\mathrm{film}}=\left(76.5\pm 9.0\right)\min^{-1}$ and $t_{0,\mathrm{film}}=\left(31\pm 4\right)\min$, was found to provide the best fit to the data sets for HPMC films from 1.8% up to 82% with the determination coefficient $R^{2}=0.98$. The griseofulvin release rate from the films then began to decline. The griseofulvin release from suspensions was found to follow the zero-order kinetics from 15% up to 67% with $K_{\mathrm{susp}}\approx 526\min^{-1}$, $t_{0,\mathrm{susp}}\approx 2\min$, $R^{2}=0.98$ and then declined. These measurements demonstrate that the encapsulation of griseofulvin nanoparticles in the porous HPMC film caused a delay of release for about 30 min until the film gradually disintegrated into small lumps [19] and also slowed its release after that. Similar delay caused by HPMC has been observed by other researchers who prepared non-porous griseofulvin-laden HPMC films via wet-casting–drying of a suspension of HPMC-griseofulvin microparticles [46]. A delay time of 7–22 min and longer delay for thicker films were reported from dissolution tests with identical equipment and dissolution medium [46]. The delayed drug release could be ascribed to the polymer swelling as well as the slow release of the drug particles from the eroding layer of the HPMC matrix. While optimizing the formulation for a specific application is not within the scope of this paper, it is possible to increase the release rate and prevent the initial delay using soluble dispersants (sugars and sugar alcohols) and superdisintegrants, which needs further investigation. Polymeric films with such low doses could be tailored for solid dosages intended for pediatric applications and/or delivery of potent drugs.

To check whether differences between griseofulvin release profiles of different specimens plotted in Figure 8 were within the acceptable limits, we computed the difference (f_1_, Equation (1)) and similarity (f_2_, Equation (2)) factors for each pair of the HPMC film and suspension specimens separately. Results of these calculations are summarized in Table S1. They demonstrate that both factors for the films and suspensions fall within the limits (f_1_ < 15 and f_2_ > 50) required for two profiles to be considered similar.

### 3.3. Drug Content and Its Uniformity

We tested the accuracy and reproducibility of loading HPMC films with seven doses of griseofulvin (0.18, 0.36, 0.73, 1.46, 2.18, 2.91, and 3.63 mg) by printing every dose on a film with individual droplets of different sizes (0.2, 0.4, 0.5, 0.8, and 1.0 µL). Depending on the dose weight, droplets were printed in arrays 1 × 5, 2 × 5, 4 × 5, 4 × 10, 6 × 10, 8 × 10, and 10 × 10 with 3-mm center-to-center separation between the adjacent droplets. We prepared three specimens of every dose and calculated the *theoretical drug content* based on the total volume of droplets printed and the suspension composition-density (refer to the top row of Table S2). We also determined the *actual drug content* and its relative standard deviation (%RSD) using the assay method in Section 2.6.3 and presented all assay results in Table S2. This test required printing 1722 droplets. To speed up printing, experiments were conducted on griseofulvin suspensions with 1.5% HPMC, having a lower shear viscosity (Figure 1).

**Figure 9:**
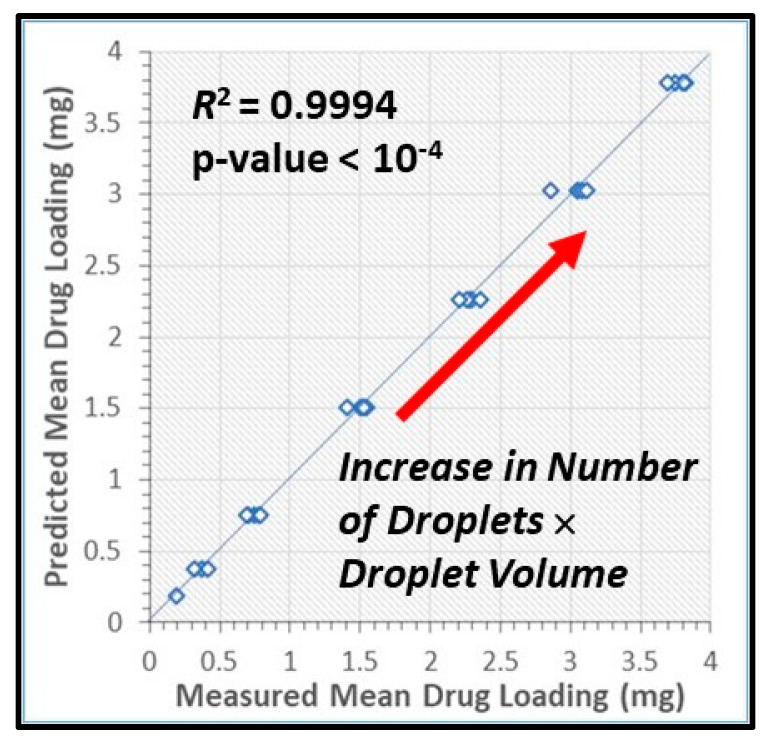
Mean drug content predicted by Equation (3) vs. the measured mean drug content in the printed films. Note that $N_{d}{\times V}_{d}$ was set at 1, 2, 4, 8, 12, 16, and 20 µL to attain 7 different levels of drug content presented in the figure. The variability observed along the horizontal direction is associated with the use of 29 different $N_{d}-V_{d}$ pairs in the experiments.

The data sets in Table S2 show that the maximum difference between the calculated (theoretical) and measured (actual) griseofulvin contents decreased rapidly from 13% for 5 droplets to below 5% with increasing the number of droplets for all droplet sizes. Figure 9 presents the mean drug content in the printed films, M, predicted by Equation (3) vs. the measured drug content:

(3) $$M\left(\mathrm{mg}\right)=189N_{d}V_{d}\left(\mathrm{mL}\right)$$

where $N_{d}$ is the number of droplets and $V_{d}$ is droplet volume. The prediction in Figure 9 is statistically significant (*p*-value < 10^−4^, 95% confidence level) with *R*^2^ = 0.9994. As the constant term in the linear regression equal to −0.0118 is statistically insignificant (*p*-value = 0.573), it was disregarded from the model equation, which is in line with the theoretical mass balance consideration. Furthermore, the proximity of the points to the 45 degree line indicates the closeness of the predicted values to the actual ones. Hence, Equation (3) establishes a certain type of “calibration curve” for setting the mean drug content in polymer films printed with the griseofulvin nanosuspension via EHD DOD. The above relationship is expected theoretically based on simple mass balance principle and the following equation:

(4) $$M\left(\mathrm{mg}\right)=\rho_{s}\varphi_{s}N_{d}V_{d}=182N_{d}V_{d}\left(\mathrm{mL}\right)$$

where $\rho_{s}$ = 1110 mg/mL and $\varphi_{s}$ = 0.164 refers to the drug nanosuspension density and mass fraction (*w/w* with respect to the suspension), respectively. Hence, the mass balance relationship, Equation (4), underpredicts the mean drug content, on the average, by 3.7%, which could be attributed to (i) sampling and analytical measurement errors, (ii) evaporation of water from the nanosuspension prior to printing, and (iii) preferential settling of some drug particles during the printing. Notwithstanding this slight deviation, Equation (4) allows for a predictive approach to dial in and vary drug content in the films without any changes to the process and the formulation. These findings signify the accuracy of the EHD DOD printing of the griseofulvin nanosuspension and its ability to impart a desired drug dose through manipulation of the number–volume of the droplets. For ~75% of the droplet number–volume cases (22/29), the films had acceptable content uniformity with RSD < 6% (Table S2). Considering the relatively low drug doses in the printed films (~0.2–4.0 mg), the achievement of such low RSD for most cases is remarkable. More importantly, for any targeted drug content studied here, the number-volume of droplets could be easily modulated to achieve acceptable drug content uniformity. In totality, these results demonstrate the ability to deposit the same amount of drug by printing a large number of small droplets or a small number of large droplets that would be useful in various applications, such as fabrication of divided drug dosages and controlled drug delivery devices.

## 4. Summary and Conclusions

We have demonstrated that the EHD DOD method [18] can be used to fabricate dosage forms by encapsulating BCS Class II drug nanoparticles into porous polymer films. Experiments were carried out on aqueous griseofulvin nanosuspensions produced by wet-stirred media milling of as-received powder to reduce the particle size from 20 µm to about 160 nm. Suspensions were printed onto an HPMC film of 0.8 mm in thickness with a porosity of 92%, whose integrity was maintained in the course of printing. Once deposited, a droplet spread over and imbibed into the HPMC film. The particles retained crystallinity and polymorphic form in the course of milling and printing. Equations used for evaluation of the EHD DOD printing regime of liquids [18] were shown to work for suspensions. Although the electrical conductivity of suspensions (0.7 mS/cm) was at the level of tap water, a high-voltage pulse did not create a short circuit between the electrodes in the course of printing, as they were not in contact with the suspension. Contrary to conventional printing technologies, a deposited droplet is pulled out from the nozzle so that the same printing regime can be utilized for a precise control over the droplet volume of suspensions with different viscosities.

Dissolution tests of six HPMC films with 50 printed droplets of 0.5 µL were conducted on a flow-through cell dissolution apparatus USP 4. Encapsulation of nanoparticles in the film caused a delay (about 30 min) and a slower release until the film gradually disintegrated into small lumps. The model-independent difference, f_1_, and similarity, f_2_, factors were calculated to demonstrate the consistency of griseofulvin release profiles from porous films with printed suspension droplets. The peak value of the griseofulvin release reached 90.5% of its content. Zero-order kinetics was found to describe the griseofulvin release rate from 1.8% up to 82%. The method versatility was demonstrated by printing the same amount of griseofulvin onto an HPMC film with droplet volume ranging from 0.2 to 1.0 µL. A difference between the measured and calculated griseofulvin contents was found to decrease rapidly from 13% for 5 droplets to below 5% with increasing the number of droplets. Overall, EHD DOD printing of the suspension of drug nanoparticles formed dosage units with good content uniformity and consistent dissolution profiles. Following the approach [27], wet-stirred media milling and EHD DOD can be easily integrated to run continuously. It should also be noted that the drug release profile can be controlled by varying the film properties (polymer type/grade, film thickness, and porosity) and the suspension formulation ingredients/composition. Various dispersants, such as sugars/sugar-alcohols and superdisintegrants, can be incorporated into either the pre-cursor drug suspensions or the films for enhancing the film disintegration and release of the drug nanoparticles. These aspects will be examined in a future study.

The presented results demonstrate the ability of the EHD DOD method to fabricate a dosage unit from an aqueous suspension of nanoparticles. Being environmentally benign and operationally safe, this printing technique offers a powerful tool for the manufacturing of precision medicines. Similar to conventional 3D printers, a 3D object can be built from thin cross sections by moving the EHD DOD head for selective layer-by-layer deposition of a liquid material.

## Figures and Tables

**Table 1:** Suspension properties at room temperature and characteristics of printing regime *.

Property	20% Griseofulvin, 1.5% HPMC, 0.5% SDS	20% Griseofulvin, 2.5% HPMC, 0.5% SDS
Density (g/cm^3^)	1.11 ± 0.02	1.12 ± 0.01
Shear viscosity (mPa·s)	32.0 ± 2.0	60.0 ± 4.0
Surface tension (mN/m)	37.0 ± 2.0	39.0 ± 1.0
Particle size, D_50_ (nm)	162.0 ± 3.0	155.0 ± 5.0
Electrical conductivity (µS/cm)	700.0 ± 5.0	690.0 ± 5.0
Dielectric constant	83.0 ± 2.0	82.0 ± 3.0
Capillary time ** (ms)	1.3	1.3
Ohnesorge number **	0.3	0.5
Bond number **	0.5	0.5
Pulse impulse **	2.8	2.8

* % refers to *w/w* with respect to DI water. ** Defined in Section 3.1.

## Supplementary Materials

The following are available online at https://www.mdpi.com/1999-4923/12/11/1034/s1: Table S1: Difference, f_1_, and similarity, f_2_, factors computed to compare griseofulvin release kinetics to one another for each pair of the HPMC film and suspension specimens. The specimen numbering refers to Figure 8a for the films and Figure 8b for the suspensions, Table S2: Griseofulvin loading (mean weight and its %RSD over three specimens) of a porous HPMC film formed by printing 1.5% HPMC-griseofulvin suspensions with droplets of different sizes.
