# Supplementary material for: Electro-Hydrodynamic Drop-on-Demand Printing of Aqueous Suspensions of Drug Nanoparticles
Source: Pharmaceutics. 2020 Oct 29;12(11):1034. doi: 10.3390/pharmaceutics12111034 (PMC7693662; doi:10.3390/pharmaceutics12111034)
Supplement: Supplementary file 1 [file pharmaceutics-12-01034-s001.pdf]

Supplementary Material: Electro-hydrodynamic Drop-on-Demand Printing of Aqueous Suspensions of Drug Nanoparticles

Ezinwa Elele, Yueyang Shen, Rajyalakshmi Boppana, Afolawemi Afolabi, Ecevit Bilgili and Boris Khusid *

Table S1. Difference, f_1, and similarity, f_2, factors computed to compare griseofulvin release kinetics to one another for each pair of the HPMC film and suspension specimens. The specimen numbering refers to Figure 8a for the films and Figure 8b for the suspensions.

Numbers		HPMC Films		Suspensions	
**Pair**	**Specimens**	**f_1**	**f_2**	**f_1**	**f_2**
1	1, 2	8.34	55.00	4.65	53.60
2	1, 3	2.22	87.11	4.14	56.60
3	1, 4	4.67	71.34	2.98	75.24
4	1, 5	6.13	66.12	2.21	73.87
5	1, 6	7.15	60.24	5.59	54.54
6	2, 3	11.03	51.94	1.65	82.49
7	2, 4	8.77	61.51	5.25	50.16
8	2, 5	4.48	70.45	3.84	59.68
9	2, 6	8.08	62.54	2.04	77.66
10	3, 4	4.65	68.09	3.91	52.91
11	3, 5	7.03	61.51	3.33	63.10
12	3, 6	7.45	57.38	1.61	85.87
13	4, 5	4.75	71.79	3.66	66.48
14	4, 6	3.88	68.84	3.74	52.10
15	5, 6	6.90	65.48	4.49	60.40
Mean		6.37	65.29	3.54	64.31
Standard deviation		2.27	8.50	1.24	11.88

Table S2. Griseofulvin loading (mean weight and its %RSD over three specimens) of a porous HPMC film formed by printing 1.5% HPMC-griseofulvin suspensions with droplets of different sizes.

Droplet Size, μL	Calculated Weight, mg	0.18	0.36	0.73	1.46	2.18	2.91	3.63
0.2	Mean weight, mg	0.19	0.37	0.74	1.52	2.28	3.05	3.80
	% RSD	4.41	1.67	1.67	4.41	4.41	4.76	4.41
	Droplet number	5	10	20	40	60	80	100
	Droplet arrangement in arrays	1 ×5	2 × 5	4 × 5	4 × 10	6 × 10	8 × 10	10 ×10
0.4	Mean weight, mg		0.41	0.78	1.55	2.29	3.05	3.82
	% RSD		12.66	7.16	6.48	4.87	4.76	4.96
	Droplet number		5	10	20	30	40	50
	Droplet arrangement in arrays		1 × 5	2 × 10	4 × 5	3 × 10	4 × 10	5 × 10
0.5	Mean weight, mg		0.32	0.70	1.50	2.26	3.08	3.74
	% RSD		12.07	3.83	3.04	3.50	5.79	2.77
	Droplet number		4	8	16	24	32	40
	Droplet arrangement in arrays		1 × 4	2 × 4	4 × 4	4 × 6	4 × 8	4 × 10
0.8	Mean weight, mg			0.79	1.52	2.36	3.11	3.81
	% RSD			8.54	4.41	8.08	6.82	4.69
	Droplet number			5	10	15	20	25
	Droplet arrangement in arrays			1 × 5	2 × 5	3 × 5	4 × 5	5 × 5
1.0	Mean weight, mg			0.69	1.41	2.21	2.86	3.69
	% RSD			5.20	3.14	1.21	1.77	1.39
	Droplet number			4	8	12	16	20
	Droplet arrangement in arrays			1 × 4	2 × 4	2 × 6	4 × 4	4 × 5
